# The complete chloroplast genome of *Torreya nucifera* (Taxaceae) and phylogenetic analysis

**DOI:** 10.1080/23802359.2019.1640091

**Published:** 2019-07-18

**Authors:** Sookyung Shin, Sang-Chul Kim, Kyung Nak Hong, Hyesoon Kang, Jei-Wan Lee

**Affiliations:** a Department of Forest Bioresources, National Institute of Forest Science, Suwon, South Korea;; b Department of Biology, Sungshin University, Seoul, South Korea

**Keywords:** *Torreya nucifera*, chloroplast genome, conifer, Taxaceae, phylogeny

## Abstract

*Torreya nucifera* (L.) Siebold & Zucc. (Taxaceae) is a tertiary relict tree species with a distribution that is limited to South Korea and Japan. In the present study, the complete chloroplast (cp) genome of *T*. *nucifera* was sequenced and analyzed. The genome was 136,985 bp in length and contained 118 genes, including 82 protein-coding genes, 33 tRNA genes, and 4 rRNA genes. Fifteen of the genes contained a single intron, whereas *ycf3* contained two introns and there were no inverted repeat sequences in the genome. Phylogenetic analysis supported the monophyly of *Torreya* species within the Taxaceae and *T*. *nucifera* was closely related to its congener *T*. *grandis*.

*Torreya nucifera* (L.) Siebold & Zucc., which is a member of the tertiary relict genus *Torreya* Arn. (Taxaceae), is a long-lived, dioecious, and evergreen gymnosperm tree species that can grow up to 25 m in height (Li and Jin 2007; Shin et al. 2017). The distribution of the species is limited to South Korea and Japan and the species is listed as Least Concern (LC) on the IUCN Red List of Threatened Species. *Torreya nucifera* was once considered an important source of wood for making furniture and Buddhist statues and the seed of species has been used as an anthelmintic medicine and for its oil (Lee 2009; Abe et al. 2016). In South Korea, *T. nucifera* exhibits a discontinuous distribution below 35°10′ N and five stands have been designated as natural monuments due to their historical and cultural importance. In the present study, the complete chloroplast (cp) genome of *T*. *nucifera* was sequenced using high-throughput sequencing technology with the aim of generating informatics data that could be used to elucidate the phylogeny of the genus and to facilitate the conservation of *T*. *nucifera*.

Fresh leaves were collected from an individual tree of *T. nucifera* Forest (Natural Monument No. 374 in South Korea) in Pyungdae-ri, Jeju-do Island (33°29′ N, 126°48′ E). Total genomic DNA was extracted using the DNeasy Plant Mini Kit (QIAGEN, Valencia, CA, USA) and subsequently stored at the Plant DNA Bank of the National Institute of Forest Science (Suwon, South Korea). Whole genome sequencing was conducted using the Ion Torrent sequencing platform (Life Technologies Corporation, Carlsbad, CA, USA) and the cp genome sequence of *T. nucifera* was assembled using Geneious R10 (Kearse et al. 2012) and the cp genome of *T. fargesii* (NCBI accession number KT027377) as a reference sequence. Annotations were assigned using NCBI BLAST searches and the tRNAscan-SE program (Schattner et al. 2005) and the complete cp genome of *T*. *nucifera* was submitted to the NCBI database (MK978775).

The complete cp genome of *T. nucifera* was 136,985 bp in length with a circular structure. The overall GC content was 35.4%. The genome contained 118 genes, including 82 protein-coding genes, 33 tRNA genes, and 4 rRNA genes. Fifteen genes contained a single intron, whereas *ycf3* had two introns. The cp DNA of *T. nucifera* did not contain a pair of IRs separated by LSC and SSC regions, which is consistent with the cp genomes of other conifers (Miu et al. 2018; Ge et al. 2019).

A maximum-likelihood phylogenetic tree was constructed using 81 protein-coding gene sequences from 13 gymnosperm taxa, with *Podocarpus lambertii* Klotzsch ex Endl. (NC_023805) and *Taxodium distichum* (L.) Rich. (LC177556) as outgroup taxa. The dataset was analyzed using the RAxML BlackBox web-server (Stamatakis et al. 2008) with 100 bootstrap replicates. The nine taxa that were members of the Taxaceae formed a distinct cluster and *T*. *nucifera* was closely related to *T*. *grandis* (NC_034806; Figure 1). These findings represent a valuable resource for the conservation of *T*. *nucifera*.

**Figure 1.**
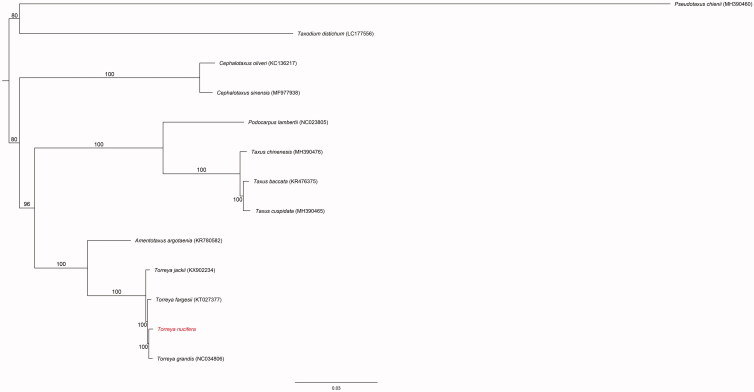
Phylogenetic position of *Torreya nucifera* inferred by maximum likelihood (ML) tree based on 81 protein-coding genes from 13 taxa. Numbers above the branch indicate the bootstrap values.
